# Optical Coherence Tomography Angiography for the Evaluation of Retinal and Choroidal Vasculature in Retinitis Pigmentosa: A Monocentric Experience

**DOI:** 10.3390/diagnostics12051020

**Published:** 2022-04-19

**Authors:** Fabrizio Giansanti, Giulio Vicini, Andrea Sodi, Cristina Nicolosi, Lavinia Bellari, Gianni Virgili, Stanislao Rizzo, Daniela Bacherini

**Affiliations:** 1 Eye Clinic, Neuromuscular and Sense Organs Department, Careggi University Hospital, 50134 Florence, Italy; fabrizio.giansanti@unifi.it (F.G.); andreasodi2@gmail.com (A.S.); cristina.nicolosi@unifi.it (C.N.); gianni.virgili@unifi.it (G.V.); daniela.bacherini@gmail.com (D.B.); 2 Department of Neurosciences, Psychology, Drug Research and Child Health, University of Florence, 50139 Florence, Italy; bellarilavinia@gmail.com; 3 Ophthalmology Unit, Catholic University of the Sacred Heart, Fondazione Policlinico Universitario A. Gemelli, 00168 Rome, Italy; stanislao.rizzo@gmail.com; 4 Consiglio Nazionale delle Ricerche (CNR), 56124 Pisa, Italy

**Keywords:** retinitis pigmentosa, OCTA, optical coherence tomography angiography, vascular density

## Abstract

Purpose: We investigated the chorioretinal microvascular changes in patients with retinitis pigmentosa (RP) by optical coherence tomography angiography (OCTA). Methods: Twenty-six patients (52 eyes) affected by RP were compared with 19 healthy controls (38 eyes). OCTA 3 mm × 3 mm macular scans were performed in all subjects. We evaluated the vessel density (VD) of the superficial capillary plexus (VD SCP), deep capillary plexus (VD DCP), choriocapillaris (VD CC), and choroid (VD choroid). We also evaluated the foveal avascular zone (FAZ) area, and the correlation between clinical and OCTA parameters. We also measured central retinal thickness (CRT) and subfoveal choroidal thickness (CT). Results: RP patients compared to healthy controls showed significantly lower VD SCP values (27.56% ± 15.37 vs. 49.39% ± 1.55; *p*-value < 0.0001), lower VD DCP values (38.43% ± 15.23 vs. 3.34% ± 0.26; *p*-value < 0.0001), lower VD CC values (46.02% ± 1.293 vs. 50.63% ± 0.4274; *p*-value = 0.0040), and lower VD choroid values (38.48% ± 15.23 vs. 3.34% ± 0.26; *p*-value < 0.0001). Even the FAZ area was significantly lower in RP patients (0.45 mm^2^ ± 0.35 vs. 0.26 mm^2^ ± 0.13; *p*-value < 0.0001). The FAZ area was larger with increasing age, both in control (r = 0.42; *p* = 0.012) and RP group (r = 0.46; *p*-value = 0.009). In RP patients, there was a statistically significant correlation between best-corrected visual acuity and VD SCP (r = 0.24, *p*-value = 0.04) and VD DCP (r = 0.52; *p*-value = 0.0004) and between subfoveal choroidal thickness and VD SCP (r = 0.43, *p*-value < 0.001) and VD DCP (r = 0.35, *p*-value < 0.001). Conclusions: In our study, OCTA reported relevant vascular alterations in RP patients in comparison with the healthy controls, in agreement with the published literature. These abnormalities were associated with choroidal atrophy and related to visual acuity loss. OCTA provided clinically significant information and may represent a reliable tool for the management of RP patients.

## 1. Introduction

Retinitis pigmentosa (RP) comprises a broad and heterogeneous group of inherited retinal dystrophies, characterized by the progressive deterioration of the photoreceptor cells and retinal pigment epithelium, which can lead to severe visual impairment [1]. The estimated prevalence of RP is 1 in 3000–5000 individuals [2].

RP typically manifests with nyctalopia during adolescence, followed by a concentric visual field constriction, reflecting the principal dysfunction of rod photoreceptors; central vision loss occurs later in life due to secondary degeneration of the cones [1,3]. The typical fundoscopic findings include mid-peripheral, bone spicule-like, pigment deposits, waxy pallor of the optic nerve head, and vascular attenuation. An electroretinogram shows a photoreceptor function markedly reduced or even absent. Optical coherence tomography (OCT) and fundus autofluorescence imaging show a progressive loss of outer retinal layers and altered lipofuscin distribution with characteristic patterns.

A precise evaluation of vascular changes in RP patients may be very useful for research and clinical purposes, in order to obtain a better comprehension of the disease physiopathology; for early diagnosis; progression monitoring; selection of patients for clinical trials; and evaluation of the possible response to innovative treatments. In particular, optical coherence tomography angiography (OCTA), a non-invasive and rapid imaging method that allows the visualization and quantification of retinal and choroidal circulation, has been recently employed in different studies to assess the microvascular changes in RP, demonstrating to be an objective, repeatable, and reliable method [4,5,6,7,8,9,10,11,12,13,14]. 

In the present research, we evaluated with OCTA the quantitative changes in chorioretinal vasculature in a group of RP patients regularly followed at the Eye Clinic of the Careggi University Hospital in Florence.

## 2. Materials and Methods

We performed an observational retrospective cross-sectional study. The study was conducted on 26 unrelated patients affected by RP, recruited through the Hereditary Retinal Degenerations Referring Center of the Eye Clinic, Careggi University Hospital, in Florence (Italy) between March 2016 and April 2017, and a control group of 19 healthy subjects. The study adhered to the tenets of the Declaration of Helsinki, and was approved by the Institutional Review Board of Careggi University Hospital. All patients signed a written informed consent, agreeing to participate.

Inclusion criteria were the diagnosis of RP, along with clear media to allow adequate OCTA-examination. Criteria for the RP phenotype included the following: (1) history of nyctalopia; (2) peripheral visual field constriction; ophthalmoscopic findings: (3) bone spicule-like pigment clumping; (4) vessel attenuation; (5) waxy pallor of the optic disc; (6) marked reduced or non-recordable a- and b-wave amplitudes on electroretinogram testing. In each case, the clinical and imaging-based diagnosis was confirmed by genetic characterization. Healthy subjects, without significant ocular pathologies or systemic disease, recruited as volunteers, acted as a control group.

Patients underwent a complete ophthalmic examination, including best-corrected visual acuity (BCVA) evaluation expressed in logMAR, intraocular pressure measurement with applanation tonometry, ocular eye examination with biomicroscopy of the anterior segment and dilated fundus examination, spectral domain OCT (SD-OCT), and OCTA. Exclusion criteria consisted of high refractive errors (myopia > 5 diopters, hypermetropia or astigmatism > 3 diopters), ocular pathologies, and opacity of dioptric means such as to prevent a good visualization of the eye fundus. We excluded patients with significant systemic diseases, with particular attention to diabetes and cardiovascular disorders, smokers (over 10 cigarettes/day), and patients who used medications that could affect the circulatory system.

We used the RS-3000 Advance SD-OCT angiographer device (NIDEK Co. Ltd., Gamagori, Japan) for capturing high-definition images at different layers. The device has a scan speed of 53,000 A-scans/s, and incorporates an eye-tracing function, to maintain the same scan location on the SLO image, despite involuntary eye movements, for an accurate image capture. It gave us the possibility to obtain a quantitative measure in terms of vascular and flow density in mm^2^. All OCTA scans were performed under full pharmacological mydriasis obtained with tropicamide drops, 1% instillation. For each eye, 3 mm × 3 mm fovea-centered scans were acquired and used for all analyses. Automated segmentation of superficial capillary plexus (SCP), deep capillary plexus (DCP), and choriocapillaris (CC) and choroid was performed. Manual adjustment of the segmentation was performed in cases of alteration of the macular cytoarchitecture. Images were reviewed by the investigators (G.V.; D.B.) for segmentation accuracy. We included in the study only high-quality scans (signal strength index of at least 9), with a mean signal strength index 9.3/10. We excluded poor-quality scans, and scans with incorrect segmentation or motion artifacts. 

**Figure 1:**
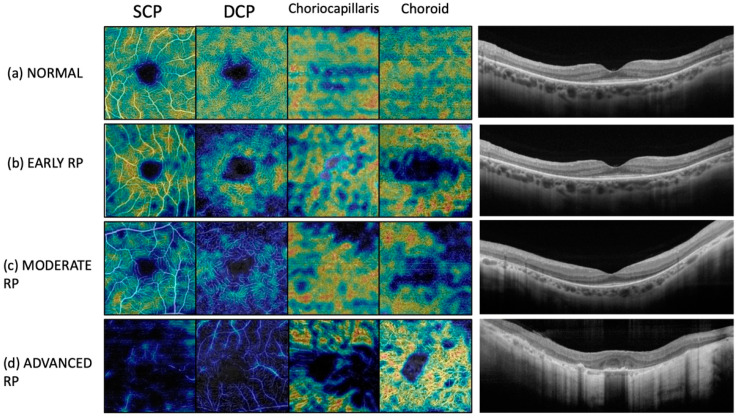
Vascular network differences between retinitis pigmentosa patients and a healthy subject in 3 mm × 3 mm OCTA scans at the (**a**) superficial capillary plexus (SCP), (**b**) deep capillary plexus (DCP), (**c**) choriocapillaris, and (**d**) choroid. In these angiograms, reduction in the vascular network density is recognizable. B-scan OCT images at different disease stages are shown on the right.

For every eye included in the study, we performed a quantitative evaluation. Vascular densities of SCP, DCP, CC, and choroid were evaluated by default device software. Vascular density was defined as the percentage of the sample area occupied by vessel lumens following binary reconstruction of images (ETDRS-based VD [%]). The FAZ area was manually measured at SCP level through the free-hand selection tool, and its dimension was expressed as square millimeters. Figure 1 shows OCTA scans in a healthy control and in patients with RP at different disease stages.

B-scan OCT quantitative measurements were obtained for all the eyes included in the study. We manually measured central retinal thickness (CRT), subfoveal choroidal thickness (CT), and CT at 1000 microns from the fovea in nasal and temporal sectors, using the software analysis tool.

Statistical analysis was performed using STATA software version 15.1 (StataCorp. College Station, TX, USA). They included descriptive statistics used to summarize demographics and main clinical records (mean values and standard deviations of all numerical data of the two groups) and comparative analysis with two-tailed Student’s *t*-test or Chi-square test with 95% confidence intervals. Spearman correlation coefficient was used as a non-parametric method to evaluate the correlation among OCTA and demographic/clinical variables. The chosen level of statistical significance was *p*-value < 0.05.

## 3. Results

### 3.1. Demographic and Clinical Characteristics

Twenty-six patients (52 eyes) affected by RP were enrolled for the study. In this group, the mean age was 50.48 ± 16.13 years. Thirteen patients (50%) were male, and 13 (50%) were female. Three of 26 RP patients were affected by Usher’s syndrome; the remaining twenty-three had non-syndromic RP.

The control group consisted of 19 healthy volunteers (38 eyes), with a mean age of 43.22 ± 15.71 years. Six patients (31.6%) were male, and 13 (68.4%) were female. 

The mean BCVA (logMAR) of the RP group was 0.18 ± 0.25. In the control group, BCVA was 0.00, and it was significantly higher in comparison with the RP group (*p*-value = 0.001). The mean intraocular pressure was 13 mmHg in both the RP and control group. No RP patients had significant media opacities and dilated fundus examination showed no other alterations except RP. Anterior segment and dilated fundus examination were unremarkable in both eyes of all controls. 

The demographic characteristics of RP patients and controls are summarized in Table 1.

### 3.2. B-Scan Optical Coherence Tomography Quantitative Measurements

We obtained B-scan OCT quantitative measurements for all the eyes included in the study. These data are resumed in Table 2. 

There were no statistically significant differences in CRT value between the RP group and control group (192.31 ± 67.29 μm vs. 191.73 ± 25.28 μm; *p*-value = 0.688). The mean subfoveal CT value was 211.82 ± 66.46 μm in the RP group and 292.92 ± 52.88 μm in the control group, and the difference was statistically significant (*p*-value < 0.0001). The mean CT value measured at 1000 microns from the fovea on the nasal sector was 195.96 ± 66.29 μm in the RP group and 261.73 ± 52.76 μm in the control group, and the difference was statistically significant (*p*-value = 0.001). The mean CT value measured at 1000 microns from the fovea on temporal sector was 216.45 ± 65.94 μm and 271.81 ± 54.05 μm, respectively, in the RP and control group, with a statistically significant difference (*p*-value = 0.007). 

### 3.3. Optical Coherence Tomography Angiography Results

Our OCTA analysis showed in RP patients a rarefaction of the vascular texture in all layers. In particular, the RP group showed, in comparison to healthy subjects, a significantly lower vascular density in SCP (27.56% ± 15.37 vs. 49.39% ± 1.55; *p*-value < 0.0001), in DCP (38.43% ± 15.23 vs. 3.34% ± 0.26; *p*-value < 0.0001), in CC (46.02% ± 1.293 vs. 50.63% ± 0.4274; *p*-value = 0.0040), and in choroid (38.48% ± 15.23 vs. 3.34% ± 0.26; *p*-value < 0.0001). We also identified a significantly lower FAZ area in RP patients compared to controls (0.45 mm^2^ ± 0.35 vs. 0.26 mm^2^ ± 0.13; *p*-value < 0.0001). A complete list of the results of this analysis is reported in Table 3. 

**Figure 2:**
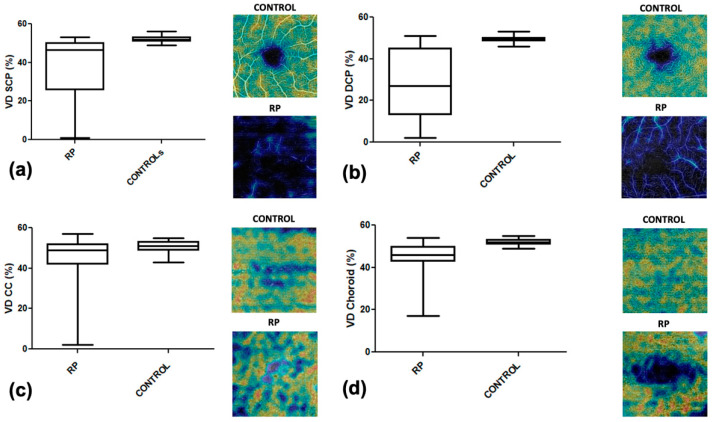
Box plots showing the relationship between controls and retinitis pigmentosa patients of the vascular density in the (**a**) superficial vascular plexus, (**b**) deep capillary plexus, (**c**) choriocapillaris, and (**d**) choroid. Statistically significant differences were observed at all layers.

**Figure 3:**
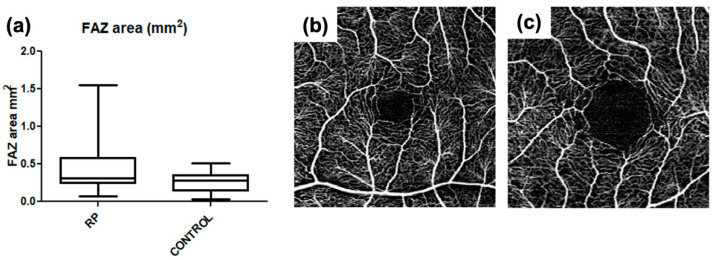
(**a**): Box plot showing the relationship between controls and retinitis pigmentosa patients, of the foveal avascular zone area. (**b**,**c**): Foveal avascular zone differences between two retinitis pigmentosa patients of different ages. The figures show the 3 mm × 3 mm OCTA of the superficial capillary plexus of two retinitis pigmentosa patients of 12-year-old (**b**) and 59-year-old (**c**), respectively. The foveal avascular zone enlargement is evident in the older patient.

**Figure 4:**
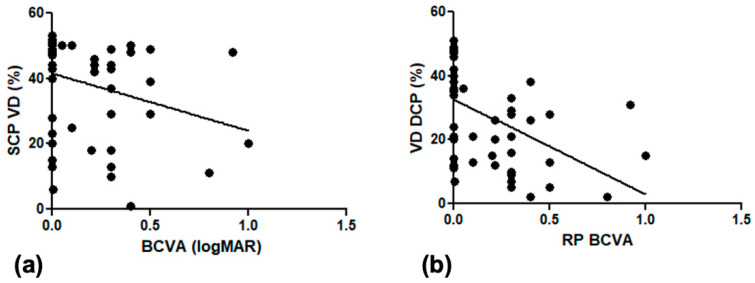
Scatterplots showing the relationship between vascular density in superficial capillary plexus (**a**) and deep capillary plexus (**b**) and best-corrected visual acuity (BCVA), expressed in LogMAR, in retinitis pigmentosa patients.

Figure 2 show box plots of the relationship between the vascular density of controls and RP patients, respectively, in SCP, DCP, CC, and choroid. In our series, the FAZ area was larger with increasing age, both in the control (r = 0.42; *p*-value = 0.012) and RP group (r = 0.46; *p*-value = 0.009), with a statistically significant correlation in both groups (Figure 3). In the RP group, patients with lower visual acuity had lower vascular density, with a statistically significant correlation between BCVA and VD in the SCP (r = 0.24, *p*-value = 0.04) and VD in the DCP (r = 0.52 *p*-value = 0.0004) (Figure 4).

In RP patients, we also found a statistically significant correlation between subfoveal choroidal thickness and VD in the SCP (r = 0.43, *p*-value < 0.001) and in the DCP (r = 0.35, *p*-value < 0.001).

## 4. Discussion

Several studies recently conducted on patients affected by RP with OCTA have reported a reduction of the retinal and choroidal blood flow, when compared to controls, and an increase in size of the foveal avascular zone (FAZ) [7,8,9,12]. Ling et al. [13] conducted a meta-analysis of the relevant published studies on OCTA in RP patients, showing that both retinal and choroidal vessels were attenuated in RP patients when compared with controls. Additionally, they revealed that the FAZ was larger and foveal thickness was smaller in RP patients compared with controls, suggesting that these microvascular parameters may have significant value in the diagnosis and monitoring of disease progression. Jauregui et al. [15] examined the OCTA progression over time and found that perfusion density decreased significantly at the superficial and deep capillary plexus and FAZ area increased significantly at the superficial and deep layers.

The reduction of retinal blood flow appeared to be significantly correlated to visual function in RP patients [6,11]. A recent study conducted by means of widefield swept source OCTA showed an impairment of retinal and choroidal perfusion density and vessel length density in both the central and peripheral retina of RP patients, if compared to healthy controls [16]. In this study, the reduction of flow features correlates with the macular function assessed with microperimetry. 

We reported the results of our Centre experience in the evaluation of retinal and choroidal microvasculature in RP patients, by means of OCTA. All the chorioretinal image acquisitions were performed with the same tool. The analysis of the data obtained in our series allows some considerations. In our series, CRT did not show a statistically significant difference between RP and controls, and this result can be explained as the RP group includes cases with macular atrophy (reduced CRT), as well normal macula until the very late stages (normal CRT) or even patients with increased CRT due to macular edema. On the other side, CT was reduced in RP for all the considered locations (subfoveal and at 1000 microns from the fovea in nasal and temporal sectors) and this is in agreement with previous studies, showing a severe or moderate choroidal atrophy in RP [17].

The quantitative evaluation of OCTA parameters in 3 mm × 3 mm macular scans showed significant differences in vascular density between RP and healthy controls at all the evaluated layers (SCP, DCP, CC, choroid), with lower values in RP patients.

The FAZ area, evaluated at SCP level, resulted significantly larger in the RP group in comparison with the controls. In both the RP and control group, the FAZ area was related to age, being wider in older patients, while the VD SCP and VD DCP in the RP group were related with BCVA and subfoveal choroidal thickness. Our data were in agreement with the published OCTA studies, confirming the vascular impairment already reported in RP.

The pathogenesis of this vascular impairment is still a matter of discussion. The rarefaction of the vascular density may be secondary to photoreceptors loss, because of the lower metabolic demand. Another hypothesis suggests a primary role of vascular supply abnormalities in the physiopathology of the disease. Moreover, an abnormal retinal vascular supply may be related to the reported choroidal alterations in RP. The retinal and choroidal microvascular alterations documented with OCTA have prompted the formulation of hypotheses on the pathogenesis of the disease such as the theory of Lu et al. [14] on the relevant role of the microglial activation and vascular dysfunction in the entire process of retinal degeneration at the base of RP. In the human eye, there is a close interaction between the choroid and the outer retinal layers, as the choroid normally releases nutrients necessary for retinal metabolism, while the RPE normally provides trophic factors promoting choroidal survival.

The positive correlation between FAZ area and age both in RP and healthy controls is not unexpected, as increasing age is reasonably associated with a rarefaction of the vascular density. On the other side in RP, the positive correlation between VD SCP and VD DCP and BCVA suggests an influence of vascular abnormalities on visual loss progression in RP. The mechanism of this influence is quite unclear and we can only speculate that even in RP, the photoreceptors’ damage is primarily genetic; a better vascular supply may play a favorable role on outer retina metabolism, then supporting a longer preservation of visual capabilities.

We are aware that our study presents some limitations. The first limitation is the small sample size; nevertheless, it should be considered the rarity of the disease. Additionally, our cross-sectional study includes a heterogeneous group of patients affected by RP, without a stratification of the disease severity, and no analyses have been made on OCTA parameters in relation to the disease degree. This is another limitation of the study. Several factors can affect OCTA results, such as diabetes, systemic hypertension, cognitive status, axial length, and signal strength of scans. We tried to overcome this limitation by excluding from the study patients with concomitant systemic diseases, such as diabetes, arterial hypertension, neurodegenerative diseases, high myopic and hyperopic patients, patients with poor quality scans, incorrect segmentation, or motion artifacts. However, it is difficult to eliminate all the possible confounding factors that could affect OCTA parameters, and this is also a limitation of the study. Lastly, the detection of vessel density in the choroid is difficult with SD-OCTA and has limitations [18,19]. However, SD-OCTA allows a vessel density detention in the choroid, although we are aware of the limitations of the method.

## 5. Conclusions

In conclusion, our study OCTA reported relevant vascular alterations in RP patients in comparison with healthy controls. These abnormalities were associated with choroidal atrophy and were related to central visual loss. If the observation of a vascular impairment in RP is further confirmed, it might promote the research of additional therapeutical approaches, aiming to improve the retinal and choroidal blood supply. In our RP series, OCTA provided clinically significant information, and may represent a reliable tool for the management of these patients, in particular for an early diagnosis, a prognostic evaluation, patients selection for clinical trials, and monitoring disease progression. 

## Figures and Tables

**Table 1:** Demographic and clinical characteristics of patients with retinitis pigmentosa and controls.

	RP Group	Healthy Controls	*p*-Value
**Number of patients (eyes)**	26 (52)	19 (38)	
Age (years) *	50.48 ± 16.13	43.22 ± 15.71	0.004 ^a^
**Gender (Male/Female)**	13 (50%)/13 (50%)	6 (31.6%)/13 (68.4%)	0.217 ^b^
BCVA (logMAR) *	0.18 ± 0.25	0.00 ± 0.00	0.001 ^a^

* Each value is expressed in mean ± standard deviation. RP = retinitis pigmentosa; BCVA = best-corrected visual acuity. ^a^ Student’s *t*-test. ^b^ Chi-square test.

**Table 2:** B-Scan optical coherence tomography quantitative evaluation.

	RP Group *	Healthy Controls *	*p*-Value
**CRT**	192.31 ± 67.29	191.73 ± 25.28	0.688
**CT subfoveal**	211.82 ± 66.46	292.9 2 ± 52.88	<0.0001
**CT nasal**	195.96 ± 66.29	261.73 ± 52.76	0.001
**CT temporal**	216.45 ± 65.94	271.81 ± 54.05	0.007

* Each value is expressed in mean ± standard deviation. RP = retinitis pigmentosa; CRT = central retinal thickness; CT = choroidal thickness.

**Table 3:** Differences in OCTA parameters between patients with retinitis pigmentosa and healthy controls.

OCTA Parameter	RP Group *	Healthy Controls *	*p*-Value
**VD SCP (%)**	27.56 ± 15.37	49.39 ± 1.55	<0.0001
**VD DCP (%)**	38.43 ± 15.23	3.34 ± 0.26	<0.0001
**VD CC (%)**	46.02 ± 1.293	50.63 ± 0.4274	0.0040
**VD choroid (%)**	38.48 ± 15.23	3.34 ± 0.26	<0.0001
**FAZ area (mm^2^)**	0.45 ± 0.35	0.26 ± 0.13	<0.0001

* Each value is expressed in mean ± standard deviation. RP = retinitis pigmentosa; VD = vascular density; SCP = superficial capillary plexus; DCP = deep capillary plexus; CC=choriocapillaris; FAZ = foveal avascular zone.

## Data Availability

The data presented in this study are available on request from the corresponding author. The data (original imaging) are not publicly available due to privacy issues.
